# Saucerneol Inhibits the Growth, Migration, and Invasion of Osteosarcoma Cells In Vitro and Prevents Metastasis‐Associated Osteolysis Ex Vivo

**DOI:** 10.1002/mnfr.70187

**Published:** 2025-07-20

**Authors:** Hyung‐Mun Yun, Nguyen Xuan Nhiem, SeonJu Park, Kyung‐Ran Park

**Affiliations:** ^1^ Department of Oral and Maxillofacial Pathology School of Dentistry Kyung Hee University Seoul Republic of Korea; ^2^ Institute of Marine Biochemistry Vietnam Academy of Science and Technology Hanoi Vietnam; ^3^ Metropolitan Seoul Center Korea Basic Science Institute Seoul Republic of Korea; ^4^ Honam Regional Center Korea Basic Science Institute Gwangju Republic of Korea

**Keywords:** apoptosis, bioactive food, osteosarcoma, ROS, STAT3

## Abstract

*Saururus chinensis* has traditionally been used to treat various diseases. Biologically active compounds isolated from *S. chinensis* exhibit diverse pharmacological activities. The aim of this study was to investigate the antiosteosarcoma effects of saucerneol (Sauc), a lignan, purified from the aerial parts of *S. chinensis* using in vitro and ex vivo experimental models. Sauc was purified from the methanolic extract of *S. chinensis*. It exhibited toxicity against MG63 and SJSA‐1 cells (human osteosarcoma cell lines), inducing apoptotic morphological changes and suppressing cell migration. Sauc triggered PARP cleavage and decreased the expression of antiapoptotic proteins. It also disrupted mitochondrial membrane potential and increased reactive oxygen species (ROS) generation. Proteome profiling, western blotting, and immunocytochemistry revealed that Sauc inhibited the JAK2/STAT3 pathway. Furthermore, Sauc downregulated the expression of metastasis‐associated proteins, suppressed invasion through extracellular matrix‐coated membranes, and inhibited anchorage‐independent cell growth. In an ex vivo bone organ culture, Sauc attenuated tumor‐induced osteolysis. This study demonstrated that Sauc exerts anti‐osteosarcoma effects by inducing apoptosis, inhibiting cell migration and invasion in vitro, and mitigating metastasis‐associated bone degradation ex vivo. Thus, Sauc holds promise as a protective compound in daily health supplements and a therapeutic agent against human osteosarcoma.

## Graphical abstract

Saucerneol (Sauc) is a phytochemical compound classified as a lignan, derived from *Saururus chinensis*. The JAK2/STAT3 signaling pathway is the primary target of Sauc in osteosarcoma cells. Sauc disrupts mitochondrial potential and increases reactive oxygen species (ROS), leading to apoptotic cell death. Sauc attenuates metastasis‐associated osteolysis in ex vivo bone organ cultures. Sauc may serve as promising a daily health supplement and phytomedicine.

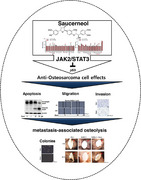

## 1 Introduction

Osteosarcoma is the most common type of malignant bone tumor, primarily affecting children and young adults of ages 10–20 years, with a secondary peak incidence observed in elderly individuals [1, 2, 3]. The pathological processes associated with primary osteosarcoma development and bone metastases involve cancer‐induced bone degradation [4]. Rapid bone growth and genetic alterations, particularly the dysregulation of the JAK2/STAT3 and p53 pathways, are associated with the development of osteosarcoma [5, 6, 7, 8]. The survival rate of patients with osteosarcoma is 70% when the tumor has not metastasized. However, this rate decreases to approximately 30% in cases where the tumor has metastasized [2, 9]. Although disease identification and monitoring have improved in recent years, the survival rates of patients and prognostic results of osteosarcoma therapy have not substantially evolved [1, 3, 10]. To address the pressing need for more effective treatments for osteosarcoma, it is essential to gain a deeper understanding of new chemotherapeutic agents as well as the cellular biology and pathology involved in the disease development process.

The perennial herb *Saururus chinensis*, found in Asian countries such as China, Japan, Korea, and Vietnam, has been used in traditional Chinese medicine [11, 12]. Lignans from *S. chinensis* have been reported to exhibit a variety of biological activities, including cardiovascular‐inhibitory, cytoprotective, hepatoprotective, bone‐protective, antiinflammatory, and anticancer activities [13, 14, 15, 16, 17]. Saucerneol (Sauc), a lignan isolated from *S. chinensis*, was recently found to exhibit potent anticancer effects. Specifically, Sauc reduces cell proliferation and metastasis in nasopharyngeal carcinoma [18]. However, its effects on other types of cancer, including sarcomas, remain unexplored. In this milieu, it is important to explore the anticancer effects and biological mechanisms of Sauc in osteosarcoma.

The objective of this study was to investigate the anticancer effects of Sauc in human osteosarcoma cells using in vitro cell culture and ex vivo bone organ culture systems, focusing on proliferation, apoptosis, migration, invasion, and tumor‐induced osteolysis. For this purpose, we purified Sauc from the air‐dried aerial parts of *S. chinensis* with a purity exceeding 99%.

## 2 Experimental Section

### 2.1 Extraction and Isolation of Sauc From the Aerial Parts *of S. chinensis*

The aerial parts of *S. chinensis* were collected from Hung Yen Province, Vietnam, in April 2024, and taxonomically identified by Dr. Nguyen The Cuong. A voucher specimen (CDRD13) has been deposited at the Institute of Ecology and Biological Resources, VAST. The air‐dried aerial parts (10 kg) were extracted three times with 80% methanol (50 L) using ultrasonication for 30 min at room temperature. After solvent removal, the methanol extract (650.0 g) was suspended in water and subsequently partitioned with dichloromethane and ethyl acetate, yielding dichloromethane (240.0 g), ethyl acetate (3.7 g), and water‐soluble (13.2 g) fractions.

The dichloromethane fraction was subjected to silica gel column chromatography, eluted with a hexane–acetone gradient (20:1 → 1:1, v/v), resulting in five subfractions: SC1A (14.4 g), SC1B (13.7 g), SC1C (22.0 g), SC1D (17.7 g), and SC1E (101.0 g). SC1E was further chromatographed on a YMC RP‐18 column with an acetone–water eluent (1.2:1, v/v) to yield two smaller fractions: SC1E1 (33 mg) and SC1E2 (288 mg). Finally, SC1E2 was purified using HPLC with a J'sphere ODS H‐80 column (250 mm × 20 mm), using 50% aqueous acetonitrile as the mobile phase at a flow rate of 3 mL/min, yielding 80.0 mg of Sauc. A stock solution (1000×) was prepared by dissolving Sauc in 100% DMSO (Sigma–Aldrich, St. Louis, MO, USA).

### 2.2 Characterization of Sauc

NMR spectra were generated using a Bruker Avance Neo 600 MHz spectrometer (Bruker, MA, USA). High‐resolution electrospray ionization mass spectrometry (HR‐ESI‐MS) was performed using a Waters ACQUITY UPLC system coupled with a Xevo G2‐XS QTOF mass spectrometer at the Metropolitan Seoul Center of the Korea Basic Science Institute (KBSI, Seoul, Republic of Korea). Preparative HPLC was performed using an AGILENT 1100 HPLC system and Sepbox 2D‐2000 at the Metropolitan Seoul Center, KBSI. CC was performed on silica gel (Kieselgel 60, 230–400 mesh; Merck, Darmstadt, Germany) and RP‐18 resins (30–50 µm; Fuji Silysia Chemical Ltd., Tokyo, Japan). Thin‐layer chromatography (TLC) was conducted on precoated silica gel 60 F_254_ plates (0.25 mm; Merck) and RP‐18 F_254S_ plates (0.25 mm; Merck).

### 2.3 Human Osteosarcoma Cell Culture

Human osteosarcoma cell lines MG63 (#CRL‐1427) and SJSA‐1 (#CRL‐2098) were purchased from the American Type Culture Collection (ATCC, Manassas, VA, USA). Using DMEM (WELGEM, Inc., Seoul, South Korea) containing 10% fetal bovine serum (Thermo Fisher Scientific, Waltham, MA, USA) and 1× Gibco antibiotic–antimycotic solution (Thermo Fisher Scientific), the cells were cultured at 37°C in a CO_2_ incubator, with an atmosphere of 95% air and 5% CO_2_, in a humidified environment.

### 2.4 3‐[4,5‐Dimethylthiazol‐2‐yl]‐2,5‐diphenyltetrazolium bromide (MTT) Assay

Cell cytotoxicity was assessed using the MTT assay (Sigma–Aldrich, St. Louis, MO, USA), which provides information on cell proliferation and viability. For this assay, 20 µL of MTT solution (5 mg/mL in PBS) was added to each well of microplates, and then the plates were incubated at 37°C for 2 h. Thereafter, 200 µL of 100% DMSO was added to each well to dissolve the formazan crystals, and the foil‐wrapped plates were incubated on an orbital shaker for 15 min. The absorbance of the samples was measured at 540 nm using a Multiskan GO Microplate Spectrophotometer (Thermo Fisher Scientific).

### 2.5 Wound Healing Assay

A 200‐µL pipette tip was used to create a scratch in the cell monolayer, allowing for the observation of cell migration [19]. Cell movement was monitored using the Olympus CKX53 inverted microscope (Olympus Corporation, Tokyo, Japan).

### 2.6 Proteome Profiling

A membrane‐based sandwich immunoassay was performed using the Proteome Profiler Human Phospho‐Kinase Array (#ARY003C; R&D Systems, USA) to simultaneously determine the relative phosphorylation levels of 37 kinase phosphorylation sites and two related total proteins without performing numerous immunoprecipitation and western blot analyses. The assay was performed in compliance with the supplier's protocol as previously described [20].

### 2.7 Western Blot Analysis

Western blot analysis was performed as described previously [21]. Briefly, the cells were washed twice with ice‐cold PBS and lysed in a lysis buffer. Protein concentration was determined using Bradford reagent (Bio‐Rad, Hercules, CA, USA). SDS‐PAGE was used to separate equal amounts of proteins (20 µg), which were then transferred onto polyvinylidene fluoride membranes (Millipore, Bedford, MA, USA). After blocking with 1× TBS containing 0.05% Tween 20 and 5% skim milk for 1 h at room temperature, the membranes were incubated with appropriate primary antibodies overnight at 4°C. After washing, the membranes were incubated with diluted secondary antibodies conjugated with horseradish peroxidase (HRP) (1:10 000; Jackson ImmunoResearch, West Grove, PA, USA) for 1 h at room temperature. The proteins were visualized using an enhanced chemiluminescence (ECL) kit (Millipore), and the signal bands in the membrane were detected using the ChemiDoc Imaging System (Bio‐Rad).

### 2.8 Information on Antibodies

The primary antibodies used in the western blot analysis and their sources are as follows: antibodies against matrix metalloproteinase (MMP)13 (1:1000, NBP1‐45723) were from Novus Biologicals (Centennial, CO, USA); antibodies against Bcl‐xL (1:500, #sc‐7195) and β‐actin (C4, 1:1000, #sc‐47778) were from Santa Cruz Biotechnology (Santa Cruz, CA, USA); and antibodies against PARP (1:1000, #9542), Bcl‐2 (1:1000, #15071), Bax (1:1000, #2772), survivin (1:1000, #2808), p‐JAK (1:1000, #3771), JAK (1:1000, #3230), p‐STAT3 (1:1000, #9145), MMP2 (1:1000, #87809), and MMP9 (1:1000, #13667) were procured from Cell Signaling Technology (Beverly, MA, USA).

### 2.9 Analysis of Mitochondrial Membrane Potential and Detection of Reactive Oxygen Species (ROS) Generation

Mitochondrial membrane potential was measured using the MT‐1 MitoMP Detection Kit (Dojindo, Tokyo, Japan), and ROS was detected using the ROS Assay Kit ‐Photo‐oxidation Resistant DCFH‐DA (Dojindo), following the manufacturer's instructions. Briefly, the cells were incubated with either 1× MT‐1 working solution or 1× photo‐oxidation resistant DCFH‐DA working solution and cultured at 37°C for 30 min in a CO_2_ incubator, with an atmosphere of 95% air and 5% CO_2_, in a humidified environment. After washing, the cells were fixed with 10% formalin solution and stained with 10 µg/mL DAPI solution (Sigma–Aldrich). The cells were observed using an intravital multiphoton microscope system (IMPM) at KBSI, and images were captured using the Olympus IX73 inverted microscope (Olympus Corporation).

### 2.10 Immunocytochemistry

The cells were seeded on eight‐well chamber slides (Thermo Fisher Scientific), fixed with 10% formalin solution, and permeabilized with 0.1% Triton X‐100 solution. After blocking the cells for 1 h at room temperature using 3% BSA blocking solution, the cells were immunostained with the anti‐p‐STAT3 (1:200; Cell signaling) antibody overnight at 4°C. After washing, the cells were incubated for 1 h at room temperature with 488‐conjugated secondary antibodies (1:500; Invitrogen, Carlsbad, CA). The nuclei were stained with 10 µg/mL DAPI solution (Sigma–Aldrich), and the cells were mounted using Fluoromount Aqueous Mounting Medium (Sigma–Aldrich) and observed with the IMPM, and images were captured using the Olympus IX73 inverted microscope (Olympus Corporation).

### 2.11 Boyden Chamber Invasion Assay

An invasion assay was performed using a Boyden chamber containing extracellular matrix (ECM)‐coated polycarbonate membranes with Matrigel solution (Corning Life Sciences, Tewksbury, MA, USA), allowing for the observation of cell invasion during metastasis, as described previously [12].

### 2.12 Soft Agar Assay

The soft agar assay was performed to observe anchorage‐independent cell growth through in vitro colony formation, as described previously [22]. On six‐well plates, 2 mL of 0.6% agar was layered at the bottom, followed by 3 mL of 0.3% agar on the top. Sauc was added to the top layer of cells. For 14 days, the cells were maintained at 37°C in a CO_2_ incubator, with an atmosphere of 95% air and 5% CO_2_, in a humidified environment. The colonies were monitored using the Olympus CKX53 inverted microscope (Olympus Corporation).

### 2.13 Ex Vivo Bone Mouse Model

Ex vivo bone organ cultures were performed to monitor metastasis‐associated osteolysis and examine the primary properties of metastatic osteosarcoma within the tumor microenvironment. ICR mice were sacrificed, and their calvarial bones were isolated for ex vivo bone organ cultures. Holes were created on each side of the calvarial bones using a biopsy punch. The calvarial bones were maintained at 37°C in a CO_2_ incubator, with an atmosphere of 95% air and 5% CO_2_, in a humidified environment using ex vivo bone culture medium. The medium was replaced every 3 days during the incubation period. The calvarial bones were observed using the Olympus CKX53 inverted microscope (Olympus Corporation).

### 2.14 Statistical Analysis

Data were statistically analyzed using GraphPad Prism version 5 (GraphPad Prism Inc., San Diego, CA, USA). All values are presented as mean ± SD. Statistical significance was determined using a one‐way analysis of variance, followed by Dunnett's post‐hoc test. Results with *p* value less than 0.05 were considered to be statistically significant.

## 3 Results

### 3.1 Isolation and Identification of Sauc From *S. chinensis*

**FIGURE 1:**
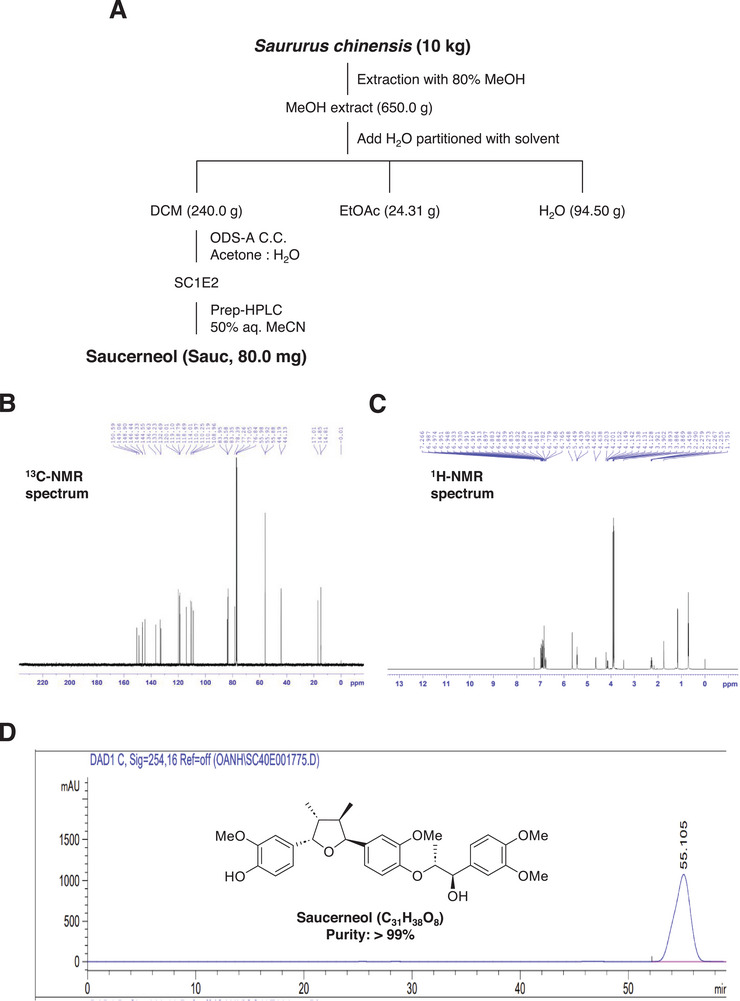
Isolation and characterization of Sauc from the aerial parts of *S. chinensis*. (A) Workflow and techniques used for isolating Sauc from *S. chinensis*. (B, C) ^13^C‐NMR (150 MHz, CDCl_3_) (B) and ^1^H NMR (600 MHz, CDCl_3_) (C) spectroscopic data for Sauc. Compound identification using HSQC, HMBC, and COSY experiments. (D) HPLC analysis of the isolated Sauc, including its chemical structure, molecular formula, and purity.

Sauc (80 mg) was isolated from the aerial parts of S*. chinensis* using the method illustrated in Figure 1A. Its identity was confirmed via NMR analysis (Table 1, Figure 1B, C). The HPLC chromatogram and chemical structure (inset) of Sauc (chemical formula: C_31_H_38_O_8_, purity: >99%) are shown in Figure 1D.

**TABLE 1:** NMR spectroscopic data for saucerneol.

C	*δ*_C_ ^a^, ^b^	*δ*_H_ ^a^, ^c^ (mult., *J* = Hz)
1	136.6	−
2	110.3	6.91 (d, 1.8)
3	150.6	−
4	146.4	−
5	118.7	6.98 (d, 7.8)
6	119.1	6.77 (dd, 1.8, 8.4)
7	83.4	5.40 (d, 5.4)
8	44.1	2.27 (m)
9	14.8	0.70 (d, 6.6)
1′	132.7	−
2′	109.0	6.84 (d, 1.8)
3′	149.1	−
4′	146.3	−
5′	114.0	6.89 (d, 8.4)
6′	118.8	6.83 (d, 1.8, 8.4)
7′	83.6	5.44 (d, 6.0)
8′	44.2	2.27 (m)
9′	14.9	0.70 (d, 7.2)
1″	133.2	−
2″	110.2	6.95 (d, 1.8)
3″	148.9	−
4″	144.6	−
5″	111.0	6.83 (d, 8.4)
6″	120.0	6.92 (dd, 1.8, 8.4)
7″	78.4	4.64 (d, 8.4)
8″	84.0	4.14 (m)
9″	17.0	1.17 (d, 6.0)
OMe	55.9	3.89 (s)
OMe	55.9	3.90 (s)
OMe	56.0	3.87 (s)
OMe	55.9	3.92 (s)

^a^ Measured in CDCl_3_.

^b^ 150 MHz.

^c^ 600 MHz, Assignments were done by HSQC, HMBC, and COSY experiments.

### 3.2 Sauc Inhibits Cell Proliferation and Induces Apoptotic Phenotypes in Human Osteosarcoma Cells

**FIGURE 2:**
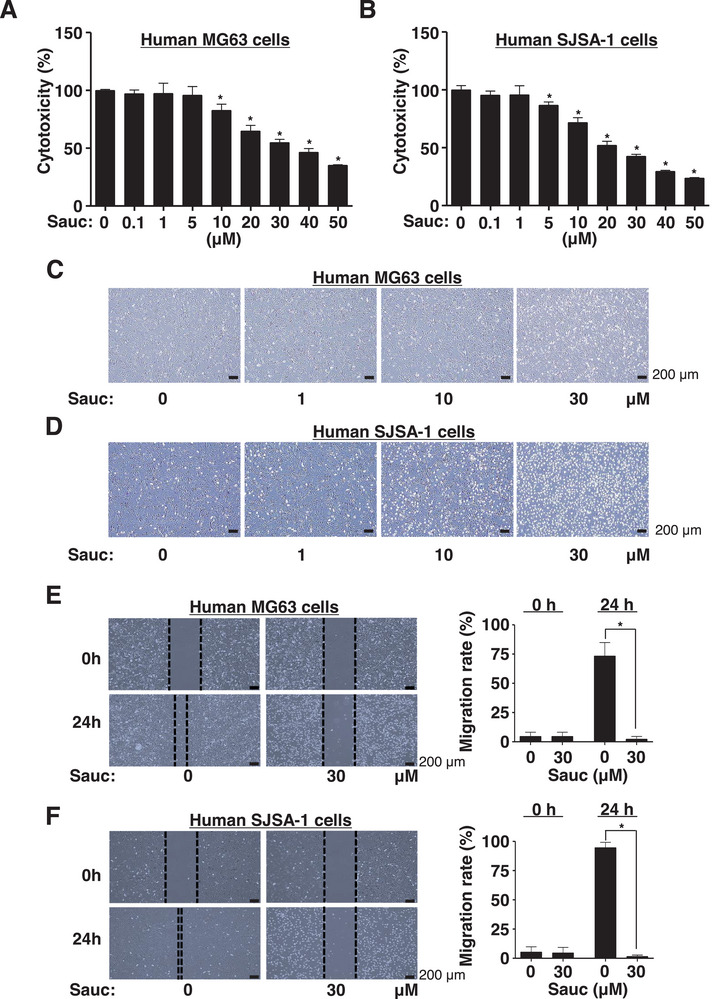
Effects of Sauc on cell death, apoptotic cell morphology, and migration in human osteosarcoma cells. (A, B) MG63 cells (A) and SJSA‐1 cells (B) were seeded in 96‐well plates and cultured with Sauc at the indicated concentrations for 24 h. The numerical values of cytotoxicity are presented as a bar graph normalized to those of the control. (C, D) MG63 cells (C) and SJSA‐1 cells (D) were seeded in six‐well plates and cultured with Sauc at the indicated concentrations for 24 h. The morphological changes were observed using the Olympus CKX53 inverted microscope. Scale bar: 200 µm. (E, F) After 24 h of Sauc treatment of MG63 cells (E) and SJSA‐1 cells (F), cell migration was assessed using the wound healing assay. The migration rate is presented as a bar graph. Scale bar: 200 µm. All values are expressed as mean ± SD of the results from three independent experiments. * Indicates a statistically significant difference, with *p* < 0.05 compared to the control.

To assess the toxicity of Sauc against human osteosarcoma cells, p53‐mutant MG63 cells and p53 wild‐type SJSA‐1 cells were treated with Sauc at the indicated concentrations for 24 h, and cell viability was analyzed. Sauc significantly reduced the viability of both MG63 and SJSA‐1 cells (Figure 2A, B); however, p53 wild‐type SJSA‐1 cells were more sensitive to Sauc than p53‐mutant MG63 cells. Sauc treatment caused both MG63 and SJSA‐1 cells to gradually shrink and transform into small spheres, which are the morphological hallmarks of apoptosis (Figure 2C, D). Additionally, Sauc inhibited wound healing in both MG63 and SJSA‐1 cells; however, distinct apoptotic morphological changes were observed in SJSA‐1 cells (Figure 2E,F). Based on these findings, we used SJSA‐1 cells, which were more sensitive to Sauc, in the subsequent experiments.

### 3.3 Sauc Induces Apoptotic Cell Death Through the Loss of Mitochondrial Membrane Potential and the Generation of ROS in Human Osteosarcoma Cells

**FIGURE 3:**
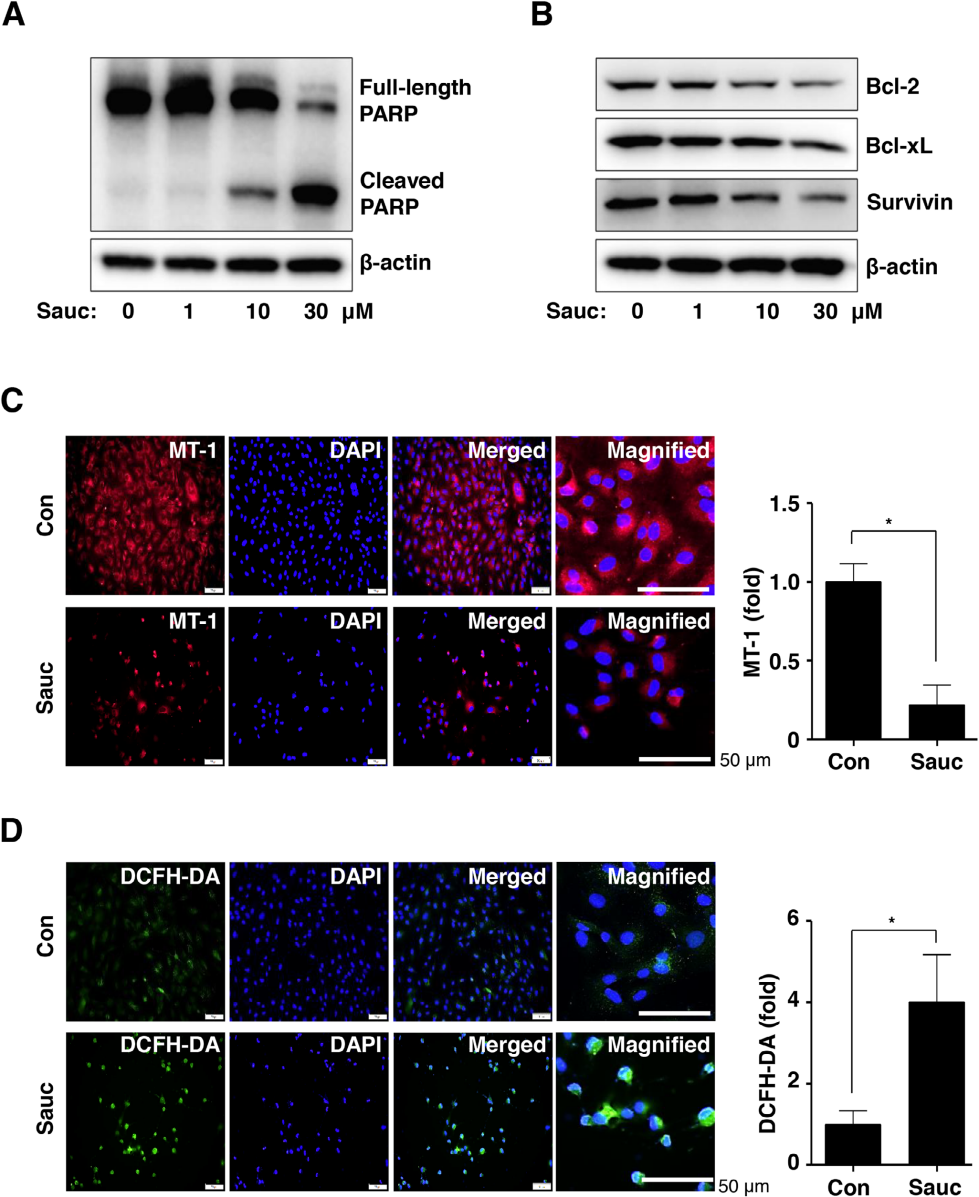
Effects of Sauc on apoptotic cell death, mitochondrial membrane potential, and reactive oxygen species (ROS) generation. (A, B) Changes in protein expression in SJSA‐1 cells were detected using equal amounts of total cell lysates after 24 h of treatment with Sauc at the indicated concentrations. PARP (A) and Bcl‐2, Bcl‐xL, survivin, and β‐actin (B) were subjected to western blot analysis. (C, D) Mitochondrial membrane potential (C) and ROS generation (D) after treatment with Sauc for 24 h were determined using MT‐1 (red) and DCFH‐DA (green), respectively. DAPI staining (blue) indicates the nuclei. The magnified images show representative cells. The relative fold changes are presented as a bar graph. Scale bar: 50 µm. All values are expressed as mean ± SD of the results from three independent experiments. * Indicates a statistically significant difference, with *p* < 0.05 compared to the control.

Based on the Sauc‐induced apoptotic phenotypes, we further evaluated the biological effects of Sauc on apoptotic cell death. Sauc increased the levels of PARP cleavage products, but decreased the level of the full‐length PARP protein in a dose‐dependent manner (Figure 3A). Next, we analyzed antiapoptotic proteins that initiate and regulate the apoptotic cell death cascade in SJSA‐1 cells. As shown in Figure 3B, Sauc reduced the expression of Bcl‐2, Bcl‐xL, and survivin (Figure 3B). We subsequently analyzed the mitochondrial membrane potential (ΔΨm) and ROS generation in SJSA‐1 cells. The accumulation of MT1 dye in the mitochondria significantly reduced following Sauc treatment compared with that in untreated cells (Figure 3C). Furthermore, the intensity of the DCFH‐DA dye increased after treatment with Sauc compared with that in untreated cells (Figure 3D). These results suggest that Sauc exerts antiosteosarcoma effects by inducing mitochondrial‐dependent apoptotic cell death, resulting in an apoptotic phenotype in SJSA‐1 cells.

### 3.4 Sauc Suppresses the JAK2/STAT3 Pathway in Human Osteosarcoma Cells

**FIGURE 4:**
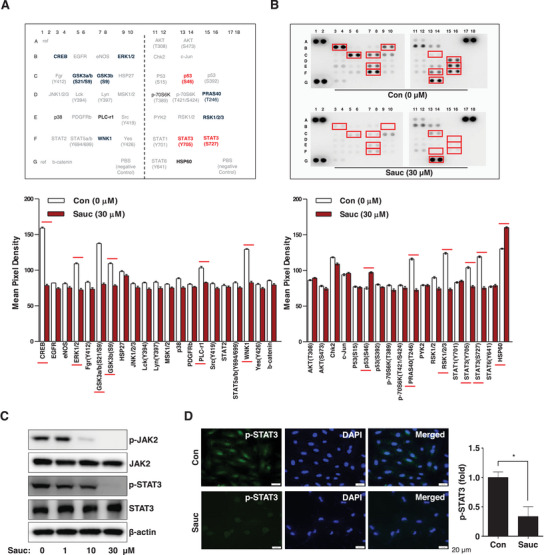
Effects of Sauc on intracellular signaling molecules. (A) The table provides details of the Proteome Profiler Human Phospho‐Kinase Array. Blue and red text highlight proteins exhibiting significant differences. (B) After 24 h of treatment of SJSA‐1 cells with Sauc, the phosphorylation profile was analyzed. Red rectangles denote double spots with substantial differences. The numerical values of the mean pixel density are presented as a bar graph. (C) Changes in the expression of p‐JAK2, JAK, p‐STAT3, STAT3, and β‐actin in SJSA‐1 cells after 24 h of treatment with Sauc, as determined using western blot analysis. (D) Immunocytochemistry was performed to assess the phosphorylation levels of STAT3 (red) following 24 h of treatment with Sauc. DAPI staining (blue) indicates the nuclei. The relative fold changes are presented as a bar graph. Scale bar: 20 µm. All values are expressed as mean ± SD of the results from three independent experiments. * Indicates a statistically significant difference, with *p* < 0.05 compared to the control.

We screened for signaling molecules involved in apoptotic cell death in SJSA‐1 cells using the Proteome Profiler Human Phospho‐Kinase Array. We found that Sauc regulated the expression of multiple intracellular proteins, including CREB, ERK1/2, GSK3b, WNK1, p53(S46), PRAS40, RSK1/2/4, STAT3(Y705), and STAT3(S727) (Figure 4A, B). In particular, the array demonstrated that Sauc decreased the phosphorylation of both STAT3(Y705) and STAT3(S727), but increased the phosphorylation of p53(S46) compared with those in nontreated cells (Figure 4A, B). The western blot analysis also demonstrated that Sauc reduced the phosphorylation of both JAK2 and STAT3 in the JAK2/STAT3 signaling pathway (Figure 4C). Furthermore, we confirmed Sauc‐induced dephosphorylation of STAT3 in SJSA‐1 cells using fluorescence microscopy (Figure 4D). These results suggest that Sauc exerts its anti‐osteosarcoma effects in SJSA‐1 cells via the JAK2/STAT3 signaling pathway.

### 3.5 Sauc Inhibits Cell Invasion, In Vitro Colony Formation, and Tumor‐Induced Osteolysis in an Ex Vivo Bone Mouse Model

**FIGURE 5:**
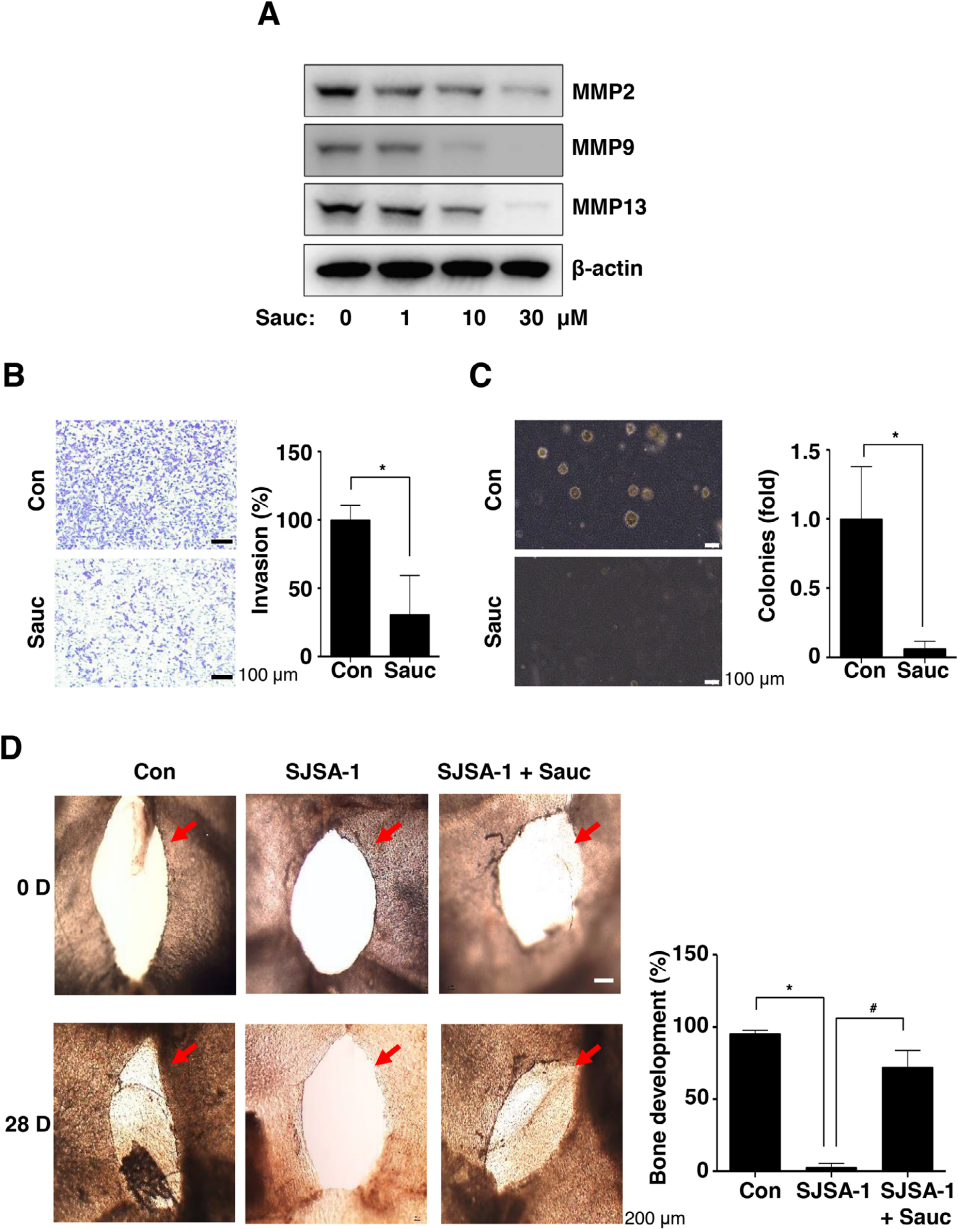
Effects of Sauc on cell invasion and colony formation, and tumor‐induced osteolysis in ex vivo bone organ cultures. (A) After 24 h of treatment of SJSA‐1 cells with Sauc, changes in the protein expression were analyzed using western blotting with antibodies against matrix metalloproteinase (MMP)2, MMP9, MMP13, and β‐actin. (B) Cell invasion was assessed using the Boyden chamber invasion assay 24 h after Sauc treatment of SJSA‐1 cells. The invasion rate is presented as a bar graph. Scale bar: 100 µm. (C) The top layer containing SJSA‐1 cells was incubated with Sauc for 14 days, and the anchorage‐independent colony‐formation ability of SJSA‐1 cells was analyzed using an inverted microscope. The colonies were counted, and the results are presented as a bar graph. Scale bar: 100 µm. (D) The bone organ cultures were divided into the following groups: control group (BMP2), SJSA‐1 cell group (BMP2 + culture media from SJSA‐1 cells), and SJSA‐1 cells + Sauc group (BMP2 + culture media from SJSA‐1 cells incubated with Sauc); they were then incubated for 28 days. The bone tissues were analyzed using an inverted microscope, and the results are presented as a bar graph. Scale bar: 200 µm. All values are expressed as mean ± SD of the results from three independent experiments. * and # indicate a statistically significant difference, with *p* < 0.05 compared to the control group and *p* < 0.05 compared to the SJSA‐1 cells + Sauc group.

We investigated whether Sauc exerts anti‐osteosarcoma effects on the tumor microenvironment. First, the expression levels of MMPs were investigated using western blot analysis. Sauc treatment decreased the expression of MMP2, MMP9, and MMP13 in a dose‐dependent manner (Figure 5A). Second, we performed a Boyden chamber invasion assay to monitor cell invasion through the degradation of the ECM during metastasis. Sauc significantly reduced the transmigration of SJSA‐1 cells across the ECM‐coated polycarbonate membrane compared with that of untreated control cells, indicating its inhibitory effect on cell invasion (Figure 5B). Next, we examined the anti‐osteosarcoma effects of Sauc on anchorage‐independent cell growth using the in vitro colony formation assay, a valuable tool for studying tumorigenicity, which is closely associated with the transformed characteristics of tumor cells. As shown in Figure 5C, Sauc significantly decreased the anchorage‐independent colony‐formation ability of SJSA‐1 cells compared with that of untreated control cells (Figure 5C). Based on the in vitro study findings, we examined the effects of Sauc on tumor‐induced osteolysis in ex vivo bone mouse model. Bone tissues developed normally in the control group; however, this process was inhibited in ex vivo bone organ cultures that contained the culture media of SJSA‐1 cells (Figure 5D). In contrast, the inhibition of bone formation was alleviated in ex vivo bone organ cultures containing the culture media of Sauc‐treated SJSA‐1 cells (Figure 5D). Overall, our results suggest that Sauc plays a crucial role in regulating ECM degradation, cell invasion, and metastasis as well as the pathological factors associated with tumor‐induced osteolysis.

## 4 Discussion

In our continuous effort to identify biologically active compounds with anti‐osteosarcoma properties [20, 23], we examined the biological effects of Sauc on human osteosarcoma MG63 cells (p53 mutant) isolated from the bone of a 14‐year‐old male patient and human osteosarcoma SJSA‐1 cells (p53 wild type) isolated from the bone of a 19‐year‐old male patient. Sauc reduced the proliferation of human osteosarcoma cells, with greater efficacy observed in SJSA‐1 cells. It is well known that apoptotic cell death is characterized by typical morphological features, including cell shrinkage and a rounded phenotype accompanied by the loss of cell adhesion. These characteristics are key indicators and are commonly used markers of apoptosis [24]. Sauc‐induced apoptotic morphological changes were more pronounced in SJSA‐1 cells than in MG63 cells. The morphological alterations associated with Sauc‐induced apoptosis were further confirmed using the wound‐healing assay. These findings suggest that Sauc may be an effective targeted treatment agent for osteosarcoma harboring p53 wild‐type genes.

To demonstrate the molecular mechanisms underlying Sauc‐mediated apoptosis in human osteosarcoma cells, we assessed PARP cleavage in human osteosarcoma cells. Our results demonstrated the cell death‐promoting effects of Sauc through the caspase cascade. Our results also demonstrated that Sauc decreases Bcl‐2, Bcl‐xL, and survivin levels in human osteosarcoma cells. Apoptosis is triggered by outer mitochondrial membrane permeabilization, which is tightly coupled with the loss of mitochondrial membrane potential, a characteristic step in the intrinsic apoptotic pathway [25]. In apoptotic cells, the loss of mitochondrial membrane potential is also accompanied by ROS generation, triggering the release of apoptogenic factors from the mitochondria [26, 27, 28]. Consistent with the previous observations, we demonstrated that Sauc induced human osteosarcoma cells to generate ROS and lose their mitochondrial membrane potential. Anticancer therapies cause oxidative stress and thereby eliminate cancer cells through ROS‐induced cell death [29, 30]. Our findings suggest that Sauc exerts anti‐osteosarcoma effects through mechanisms that induce mitochondrial‐dependent apoptotic cell death.

Under physiological and pathological conditions, signaling pathways control cell growth, apoptosis, and tumorigenesis [31, 32]. In the present study, we investigated the specific intracellular signaling pathways through which Sauc treatment causes apoptotic cell death. We found that Sauc reduced the phosphorylation of signaling molecules, including STAT3(Y705) and STAT3(S727), whereas it increased the phosphorylation of p53(S46). It has been reported that STAT3 inhibits p53‐mediated apoptosis and cell growth arrest [8, 33]. p53 causes apoptosis by permeabilizing the mitochondrial membrane by interacting with and suppressing the activity of the anti‐apoptotic proteins Bcl‐xL and Bcl‐2 [34]. Consistent with this evidence, we found that Sauc inhibits cell growth and induces apoptosis, and that p53 wild‐type SJSA‐1 cells are more susceptible to Sauc than p53‐mutant MG63 cells. The enhanced sensitivity of p53 wild‐type SJSA‐1 cells to Sauc was speculated to be related to increased p53 phosphorylation. Furthermore, we confirmed that Sauc inhibited the JAK2/STAT3 signaling pathway. The constitutively activated JAK2/STAT3 signaling pathway is closely associated with the development, progression, and poor prognosis of osteosarcoma [35]. These findings suggest that Sauc exerts anti‐osteosarcoma effects through the JAK2/STAT3 signaling pathway, although further studies are needed to fully elucidate this mechanism and its implications.

The complicated process of metastasis includes the detachment of cells from the primary site, degradation of the ECM, migration and invasion of tumor cells, and colonization of new niches [36]. In several cancers, the activation of the JAK2/STAT3 signaling pathway is strongly associated with increased invasiveness and metastasis [37, 38]. In the present study, we demonstrated that Sauc inhibits cell migration and invasion through the degradation of the ECM and inhibition of colony formation in osteosarcoma cells. Increased MMP‐2, MMP‐9, and MMP‐13 levels mediate the invasion, migration, and metastasis of osteosarcoma cells [39]. We demonstrated that Sauc suppresses the expression of MMP‐2, MMP‐9, and MMP‐13 in osteosarcoma cells. The ex vivo bone organ cultures also showed that Sauc reduces tumor‐induced osteolysis, which is consistent with the in vitro results. Bone remodeling creates an environment that supports tumor formation and metastasis in both primary osteosarcoma and bone metastases [4]. Therefore, the spread of osteosarcoma cells is significantly influenced by bone formation and resorption. These findings suggest that Sauc induces anti‐osteosarcoma effects by preventing metastatic potential and pathological bone degradation in the tumor microenvironment.

In conclusion, to the best of our knowledge, this is the first study to report that Sauc induces mitochondrial‐dependent apoptotic cell death and inhibits cell migration and invasion, and metastasis‐associated osteolysis via the JAK2/STAT3 signaling pathway. Nevertheless, the present study has limitations owing to its reliance on in vitro and ex vivo assays of Sauc. Future research should validate the anti‐osteosarcoma effects of Sauc in in vivo animal models and explore its potential for drug development. Furthermore, it is essential to investigate various types of osteosarcoma beyond the specific subtype associated with p53. The clinical feasibility of Sauc application should be assessed through the analysis of the absorption, distribution, metabolism, and excretion of Sauc. Finally, it is essential to compare the effects of Sauc with those of other anti‐osteosarcoma medications. Our study provides compelling evidence that Sauc may be beneficial as a routine health supplement or as an anti‐osteosarcoma treatment agent with further validation.

## Ethics Statement

The Animal Care Committee of Kyung Hee University granted authorization for all experimental procedures (approval number: KHSASP‐23‐294). The study adhered to the ethical guidelines established by the National Institute of Toxicological Research of the Korea Food and Drug Administration regarding the care and use of laboratory animals.

## Conflicts of Interest

The authors declare no conflicts of interest.
